# Crash analysis of mountainous freeways with high bridge and tunnel ratios using road scenario-based discretization

**DOI:** 10.1371/journal.pone.0237408

**Published:** 2020-08-10

**Authors:** Zongyuan Sun, Shuo Liu, Dongxue Li, Boming Tang, Shouen Fang

**Affiliations:** 1 Department of Traffic and Transportation, Chongqing Jiaotong University, Chongqing, China; 2 Department of Transportation Engineering, Tongji University, Shanghai, China; South China University of Technology, CHINA

## Abstract

Mountainous freeways with high bridge and tunnel ratios are a new type of road that rarely contain many special road sections formed by various structures. The crash characteristics of the road are still unclear, but it also provides conditions for studying how various road environments affect traffic. In view of the various structures and differences in the driving environments, a scenario-based discretization method for such a road was established. The traffic-influence areas of elementary and composite structures were proposed and defined. Actual data were analyzed to investigate the crash patterns in an entire freeway and in each special section through statistical and comparative research. The results demonstrate the applicability and validity of this method. The crash rates were found to be the highest in interchange and service areas, lower in ordinary sections, and the lowest in tunnels, being mostly attributed to collisions with fixtures. The crash severity on bridges and bridge groups was significantly higher than that on the other types of road sections, being mostly attributed to single-vehicle crashes. The annual average daily traffic and driving adaptability were found to be related to crashes. The findings shed some light on the road design and traffic management implications for strengthening the traffic safety of mountainous freeways.

## Introduction

Mountainous freeways with high bridge and tunnel ratios are becoming more prevalent in the middle and western regions of China owing to the rapid improvement in the economy and the implementation of the national freeway network improvement plan. For example, the Ankang section of the Xihan freeway, which has a total length of 78 km, has a bridge and tunnel ratio as high as 70%. Similarly, bridges and tunnels constitute 63% of the Chongqing section of the Yuxiang freeway, which has a total length of 270 km. This is because tunnels and bridges can not only traverse complex terrains within a short distance but also help improve traffic efficiency and reduce air pollution; they are becoming more common owing to modern construction technologies and lower construction costs. Consequently, long tunnels and bridges are replacing long roadways in mountainous freeways.

Owing to their large number of bridges, tunnels, and interchanges, these new mountainous freeways are characterized by frequent cross-section switching, abrupt changes in longitudinal driving environments, and alternating light and dark areas. This makes the entire driving process dynamic and difficult [1, 2]. These conditions are detrimental to drivers and pose a challenge to engineers striving to create continuous and safe driving conditions. Considering such complex driving environments, the ability of drivers to adapt to this new type of a road is the key issue to be considered before large-scale construction can begin. Additionally, the increasing number of bridges and tunnels has led to a higher number of crashes, including serious crashes, on many of these structures. For instance, a traffic crash occurred at the Shanxi Qinling tunnel group on August 10, 2017, when a coach collided with a tunnel entrance, resulting in 36 deaths and 13 injuries. Such serious crashes raise more concerns about the safety of this new type of a road. In particular, concerns have been raised about whether this new type of a road is more dangerous than other highways are and whether additional circumstances attributed to the presence of so many structures have contributed to the crash. The impact of various special road environments on traffic is still unknown. Conducting road traffic crash analysis can be an effective approach to solve these problems [3, 4].

Numerous crash investigations have been conducted on typical mountainous highways [5–7]. Some researchers have analyzed crash data through statistical regression models to establish crash prediction models or investigate factors contributing to crashes [8, 9]. However, this research has limited applicability to mountainous freeways with high bridge and tunnel ratios. Huang et al. [10] employed a classification and regression tree model to analyze the interactive risks related to serious car crash injuries on mountainous freeways with tunnels and found that although driving behavior, crash time, grade, curve radius, and vehicle type were significant factors, severe crashes mostly occurred owing to a combination of effects including weather and crash location. Duan et al. [11] analyzed road traffic crashes on the Yuxiang freeway and found that bridge and tunnel ratios, annual average daily traffic (AADT), and road length were the main factors affecting the number of crashes. Zhang et al. [12] applied isometric division to analyze the traffic crash data and found that 65% of crash blackspots were in tunnel groups, 30% of which were tunnel exits adjacent to interchanges or freeway service areas. Furthermore, rear-end crashes accounted for approximately 66% of the total number of crashes, followed by tunnel sidewall impacts and overturning. For tunnels and tunnel groups, several studies [13–16] investigated crashes by dividing the tunnels into different zones based on the driving environment. The researchers then compared and studied the crash characteristics of each zone, including the crash rates, severity, and type. Nevertheless, similar studies on bridges have not been conducted.

Because mountainous freeways with high bridge and tunnel ratios are relatively new, few studies have been conducted on crashes on these types of roads, and the mechanisms are still unclear. Existing research [9, 11, 15–17] has mainly focused on general mountainous freeways and has considered either the entire freeway or a specific section of the freeway, such as tunnels, tunnel groups, and bridge–tunnel groups. Few global comparative studies have been conducted, and fewer considered the various road sections formed by these structures. Furthermore, most studies still use the general road division method, and only a few studies have been conducted in which the road is divided according to the differences in operating environments.

In mountainous freeways with high bridge and tunnel ratios, many bridges, tunnels, and necessary interchanges and freeway service areas have to be constructed; therefore, they have a large number of structures, which have external influence areas outside their own physical area, and the driving environment in these influence areas are different from those on ordinary roads. In addition, when the same type or different types of structures interact, they form unique road sections such as bridge groups, tunnel groups, and bridge–tunnel groups. Therefore, compared with ordinary highways, these structures have become major components of such roads, with a significant advantage in terms of length and larger traffic influence areas. More importantly, these structures form a variety of unique road sections, each of which has its own complex traffic operations. Although these small sections create different and independent driving conditions, they coexist within the entire driving process and thus determine the overall safety performance of the road.

To investigate the crash patterns for the entire road and each type of road section, in mountainous freeways with high bridge and tunnel ratios, appropriate and accurate discretization methods are critical for exploring the differences in crash mechanisms and determining how these factors are influenced by various road sections. Therefore, this study attempts to first establish a scenario-based discretization method of the road. Based on the specific traffic scene formed by the various types of individual and composite structures, the degree of intervention in driving is comprehensively measured in terms of the driver’s visual needs and adaptations, traffic organization behavior, and road conditions. The traffic impact areas of elementary structures are proposed and determined, and the special sections for composite structures are defined by calculating the critical safety distance index. Finally, with the help of this method, the statistics and comparative analysis of actual crashes in China are obtained and conducted, respectively.

## Methodology

### Road scenario-based discretization method

#### Determination of traffic influence area of elementary structures

The mainline of mountainous freeways with high bridge and tunnel ratios comprises elementary structures including bridges, tunnels, interchanges, and service areas. In addition to having their own specific traffic operating environments, these structures have a certain external influence on areas near entrances and exits. All these areas constitute the scope of special road sections for elementary structures. The scope is defined as the zone that influences traffic—the structure body and its contiguous section that influences a driver’s normal safe driving pattern. This is often due to the road traffic specific to the structure. Although both the structure and its traffic influence area are special sections with different driving environments, the scope of the latter is significantly larger than that of the former. The influence area determines the real boundary of the influence of the structure on the driving conditions, and therefore, it is reasonable to apply it to road discretization. The entrances and exits of different elementary structures have different driving safety characteristics. Therefore, when calculating the external influence distance, it is necessary to consider the following main factors and treat them differently to determine the influence area.

1. Tunnels
  When vehicles approach the entrance of a tunnel during the day, drivers often tend to slow the car down owing to the black hole effect. According to the luminance recommendations as per the highway lighting guidelines [18], the tunnel access zone is defined as the open road immediately outside the entrance, which begins at the stopping sight distance before the tunnel entrance and ends at the entrance itself. With a driving speed of 100 km/h (which is the speed limit outside the tunnel section in mountainous freeways in China), the stopping sight distance is 150 m. When leaving the tunnel, the vehicle enters the bright outside environment, and the vision of the driver is significantly influenced by the high brightness and visual stimulation outside the tunnel. According to a previous experimental driving study on Chinese freeway tunnels [19] and the Chinese tunnel lighting guideline (JTG/T D70/2-01-2014) [20], the time required to adapt to light is typically 3–15 s depending on the length of the tunnel. According to the Chinese highway engineering technique standard (JTG B01-2014) [21], road tunnels can be divided into four categories: short tunnels (L < 500 m), medium tunnels (500 m ≤ L < 1000 m), long tunnels (1000 m ≤ L < 3000 m), and extra-long tunnels (L ≥ 3000 m). The corresponding maximum light adaptation distances are 250, 300, 350, and 400 m. Therefore, the influence of entrance and exit traffic and the distance of a tunnel are determined.
2. Bridges
  On the one hand, bridge decks are very high, which results in the driver having a certain sense that the canyon is deep and that there is a high risk of injuries and casualties in the event of a fall. On the other hand, changes in the visual environment of the bridge entrance and exit are less significant than those of tunnels. The influence on driving is relatively low, which was also demonstrated by the results of a vehicle driving test [22]. Considering these aspects, only the stopping sight distance (D_Stop_), which is directly related to the driving safety, is considered as the bridge entrance and exit influence distance.
3. Interchanges and freeway service areas
  Both interchanges and service areas have specialized traffic organization sections. Their traffic influence areas are similar and should be determined mainly according to the influence of traffic flow, which is very different from those of bridges and tunnels and is mainly affected by visual characteristics. According to the highway capacity manual (2010), the influence area of a freeway entrance begins and ends 450 m before and 150 m after the exit ramp-way junction, respectively, and that of a freeway exit begins and ends 150 m before and 450 m after the entrance ramp-way junction, respectively. In this study, the affected road sections of the entrance and exit and their connecting section are considered as the traffic influence area of an interchange and service area.

#### Definition and division of unique road sections with composite structures

When elementary structures are sufficiently close, unique road sections with composite structures are formed by a similar or heterogeneous combination such as tunnel groups, bridge groups, and bridge–tunnel groups. Although existing research has provided definitions for these unique road sections [10, 15, 23], most of them lack clear quantitative standards and bases, and a uniform view has not yet been achieved. The root of the problem lies in how the distance between structures can be determined and how these structures can be turned into a composite road section in accordance with the actual conditions. Accordingly, this article defines these sections as a road section containing two or more elementary structures spaced by a distance less than the safety critical distance.

**Fig 1:**
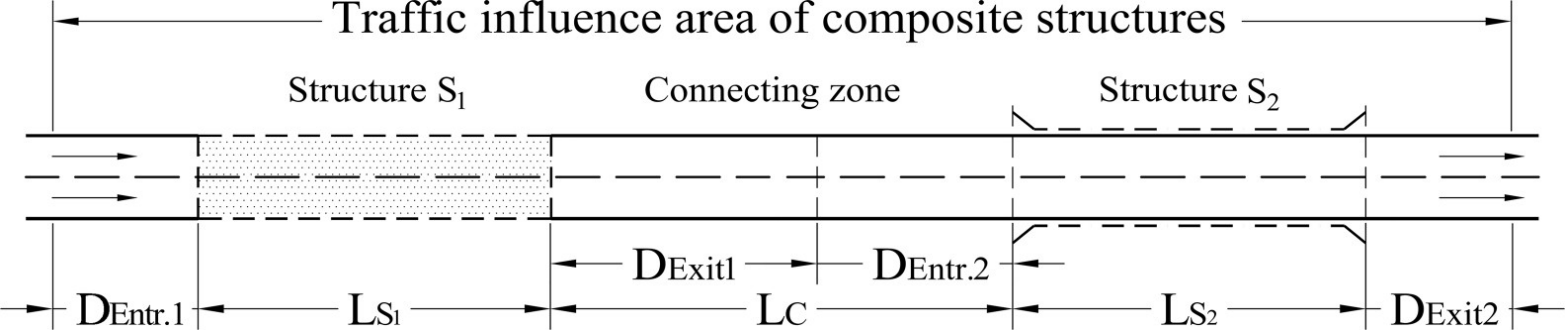
Diagram of traffic influence area of composite structures.

When defining a unique road section with composite structures, the safety-critical distance (L_C_) refers to the specific distance between them, which is determined for safety reasons. This distance is the only quantitative basis for judgment. As shown in Fig 1, S_1,_ and S_2_ are two adjacent structures. If D_Exit1_ is defined as the exit influence distance of the first elementary structure and D_Entr.2_ the entrance influence distance of the second elementary structure, then L_C_ is equal to the sum of D_Exit1_ and D_Entr.2_. For different composite structure types and orders of appearance, the values of D_Exit1_ and D_Entr.2_ are different owing to various factors, and different values of L_C_ can be obtained. If the distance between two adjacent structures (L) is less than L_C_, there are overlapping areas between the traffic influence areas of the two structures. When driving on these sections, the driving adaptation of S_1_ will affect the driving process when approaching S_2_. Hence, on a portion of the connecting section, driving will be affected by the influence of both the structures. Such adjacent structures have a compound influence on driving safety; thus, they should be treated as a composite structure instead of two single structures.

Similar to that of an elementary structure, the traffic influence area of a composite structure comprises the structures, connecting section, and contiguous road section outside the entrance and exit, as shown in Fig 1. The unique section with composite structures should also be discretized by the corresponding traffic influence area. The unique sections of the composite structures, values of the critical safety distances, and distances of the entrances and exits were calculated according to the traffic influence distances of the entrances and exits of the three elementary structures. The results are summarized in Table 1.

**Table 1:** Characteristics of traffic influence area with composite structure.

Type		The safety critical distance (m)	Traffic influence Distance (m)	
			Entrances	Exits
Tunnel groups		600	150	[250,400]
Bridge groups		300	150	150
Tunnel-bridge groups	Bridge before tunnel	300	150	[250,400]
	Tunnel before bridge	600	150	150
Tunnel-interchange (Service area) groups^a^	Entrance before tunnel	600	450	[250,400]
	Tunnel before exit	900	150	450

^a^ The following abbreviations Tunnel-interchange groups.

### Data basis and preparation

**Fig 2:**
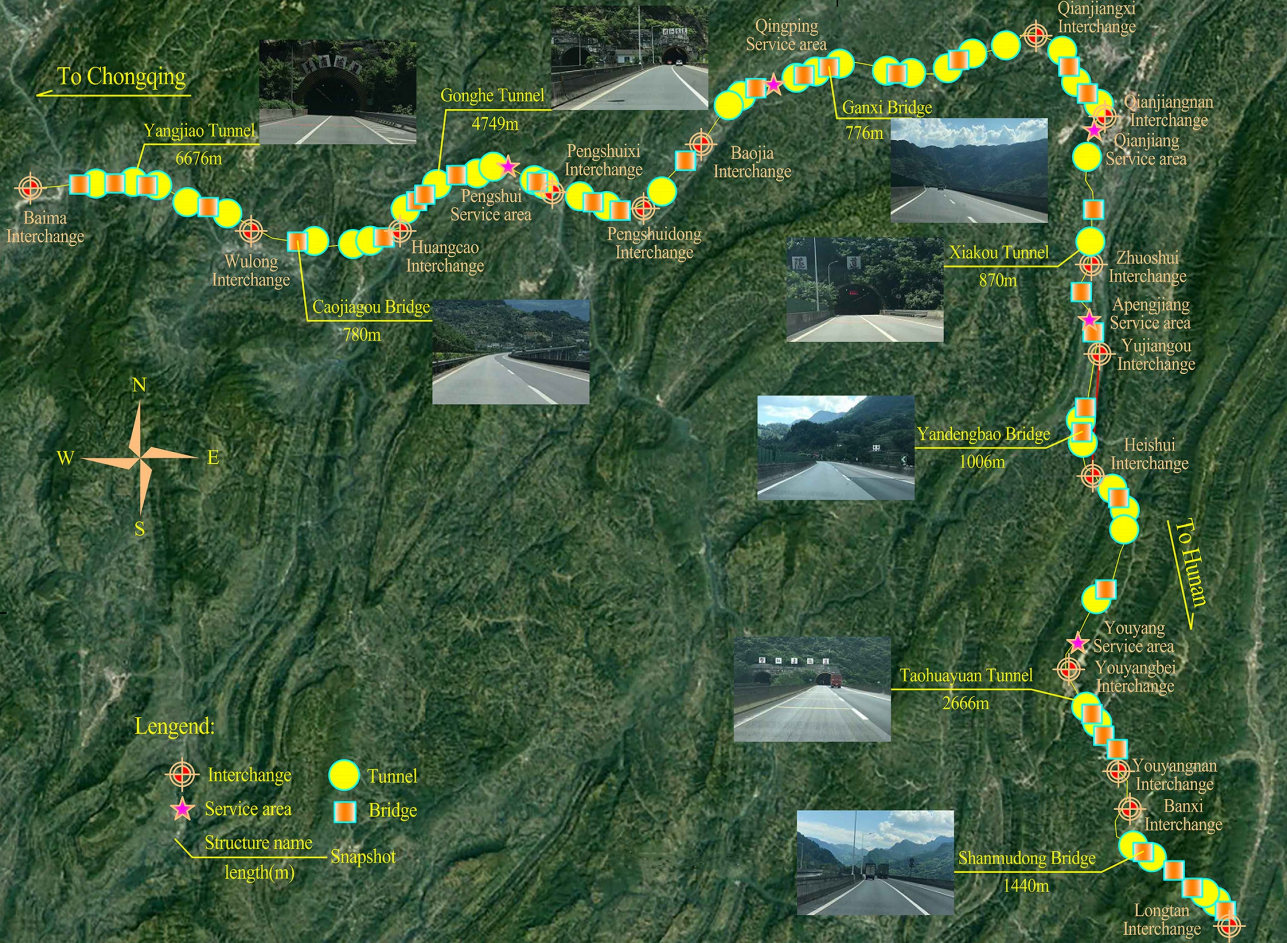
The sketch of selected road section.

The freeway section under consideration has a length of 254.12 km from Baima to Longtan and is part of the freeway connecting Chongqing and Hunan, located in the city of Chongqing, China. This selected road section passes through extremely mountainous areas and has two lanes in each direction. In this section, there are many bridges and tunnels with short separation distances, which account for 61.85% of the total length, and therefore, it is a typical mountainous freeway with a high bridge and tunnel ratio. The entire section is divided into 14 basic road sections by interchanges. Table 2 summarizes the characteristics of the structures in this road section, while Fig 2 shows the sketch of selected road section.

**Table 2:** Characteristics of structures for the road section.

Type		Number	Length (km)	Lanes	Speed limit (km/h)
Tunnels	Short	14	4.02	2	80
	Medium	11	8.26		
	Long	21	37.89		
	Extra-long	11	48.15		
Bridges	Small & Medium	24	1.75	2	100
	Large	140	46.11		
	Extra-large	4	4.69		
Interchanges		15	8.15	2	
Service areas		5	2.61	2	
Total		245	161.64	2	-

The traffic crash data were obtained from police crash records, which were provided by the Chongqing Department of Traffic Police. The records cover various aspects of a traffic crash, including demographic characteristics for casualties, crash cause, collision types, vehicle types, and environmental factors, such as weather conditions, as well as the precise time and location of the crash. During the study period between 2014 and 2016, a total of 1739 crashes occurred, of which 230 occurred on the interchanges and freeway service areas and 1509 on the remaining basic road sections, which are the mainline of the freeway. The crashes under consideration included fatality crashes, injury crashes, and property-damage-only crashes. During this three-year period, there were no notable changes in the road conditions or traffic-management strategies.

It is crucial for crashes and structures to match accurately, and a crash location is the link between them. Therefore, the locations of both the structures and crashes were extracted and checked in two manners. The positions of various structures were first extracted according to the road blueprints and determined according to the road pile number obtained through an actual driving test. For information such as the location of major crashes and the resulting casualties, the corresponding recorded road traffic crash investigation report was used to provide additional fact-checking on the basis of the crash data registration form.

## Results and discussion

As previously mentioned, based on the definition and division method for unique road sections, the selected road section was divided into eight road types: tunnels, bridges, interchanges, freeway service areas, tunnel groups, bridge groups, tunnel–bridge groups, tunnel–interchange groups, and ordinary road sections. However, a few of the junctions with structures may belong to different types of unique road sections. The corresponding unique sections were identified as discrete elements, resulting in the division of the entire road section into 324 sub-sections, of which the east and west stretches consisted of 159 and 165 sub-sections, respectively. Depending on the location, 1739 crashes were matched with their respective sections, and crashes were combined if they occurred in sections with the same road type. If crashes belonged to an overlapping area of different road types, they were counted into their corresponding road types.

### Traffic crash rate on each type of road section

The road traffic crash rate for each type of road section on the mountainous freeway with a high bridge and tunnel ratio was calculated, and the results are presented in Table 3. The vehicle-kilometer values used were determined as the sums for each road section length. For unique road sections with a single structure, the crash rate on interchanges and service areas was the highest (1.78) and was nearly six times as high as that of tunnels (0.30), whereas that of bridges was an intermediate value of 0.43. For unique road sections with composite structures, bridge groups had the highest crash rate (0.40). The crash rate of bridge–tunnel groups was the same as that of interchange–tunnel groups (0.36) and that of tunnel groups was the lowest (0.34). Therefore, the various road sections are ranked in ascending order according to their crash rate as follows: interchanges and service areas > ordinary sections > bridges > bridge groups > bridge–tunnel groups = interchange–tunnel groups > tunnel groups > tunnels. Unexpectedly, the crash rate of ordinary road sections was higher than that of the unique road sections, except at interchanges and service areas, whereas the crash rate of tunnels was the lowest.

**Table 3:** Crashes rates on the various types of road sections.

Road type	Length (km)	AADT (veh./day)	Travel (10^6^ km·veh./year)	The number of Crashes	Crashes rates (acc./10^6^ km·veh)
Tunnels	30.06	13784	151.25	134	0.30
Bridges	23.81	13634	118.47	154	0.43
Interchanges and service areas	8.40	13515	41.46	221	1.78
Tunnel groups	72.23	14880	392.31	404	0.34
Bridge groups	14.65	13345	71.34	86	0.40
Bridge-tunnel groups	80.20	14803	433.32	466	0.36
Interchange-tunnel groups	13.99	13453	68.66	74	0.36
Ordinary sections	84.31	13500	415.45	571	0.46
Entire road section	254.12	13909	1290.13	1739	0.45
Entire road section^a^	243.95	13922	1239.62	1509	0.41

^a^ Interchanges and service areas are excluded from the computation.

### Traffic crash types

To further analyze the traffic crashes on each type of road section, all crashes were divided and categorized by crash type. The results are presented in Table 4. The proportions of sideswipe collisions, rollovers, and other crashes with low frequencies on the entire road are comparable to those on ordinary road sections; the former is approximately four times higher than the latter. The proportion of rear-end crashes with the highest frequency on the entire road is approximately 10% higher than that on ordinary road sections, whereas the proportion of collisions with fixtures is the opposite of that of rear-end crashes. This indicates that it is more difficult for drivers to deal with inter-vehicle interactions on mountainous freeways with high bridge and tunnel ratios than on ordinary road sections. Among the causes of crashes, improper car-following behavior is the main cause of the high incidence of crashes.

**Table 4:** Crashes types on the various types of road sections.

Crash type	Multivehicle crashes		Single vehicle crashes		Other types
	Rear-end collisions	Sideswipe collisions	Collisions with fixtures	Rollovers	
Tunnels	52.2	13.4	24.6	3.0	6.7
Bridges	42.9	13.0	38.3	3.2	2.6
Interchanges and service areas	29.0	32.1	28.5	2.3	8.1
Tunnel groups	60.4	13.1	21.3	2.7	2.5
Bridge groups	45.3	11.6	38.4	3.5	1.2
Bridge-tunnel groups	61.4	11.2	22.3	2.6	2.6
Interchange-tunnel groups	36.5	24.3	31.1	4.1	4.1
Ordinary sections	33.3	15.9	42.7	6.5	1.6
Entire road section	43.2	16.2	32.0	3.9	4.6

**Fig 3:**
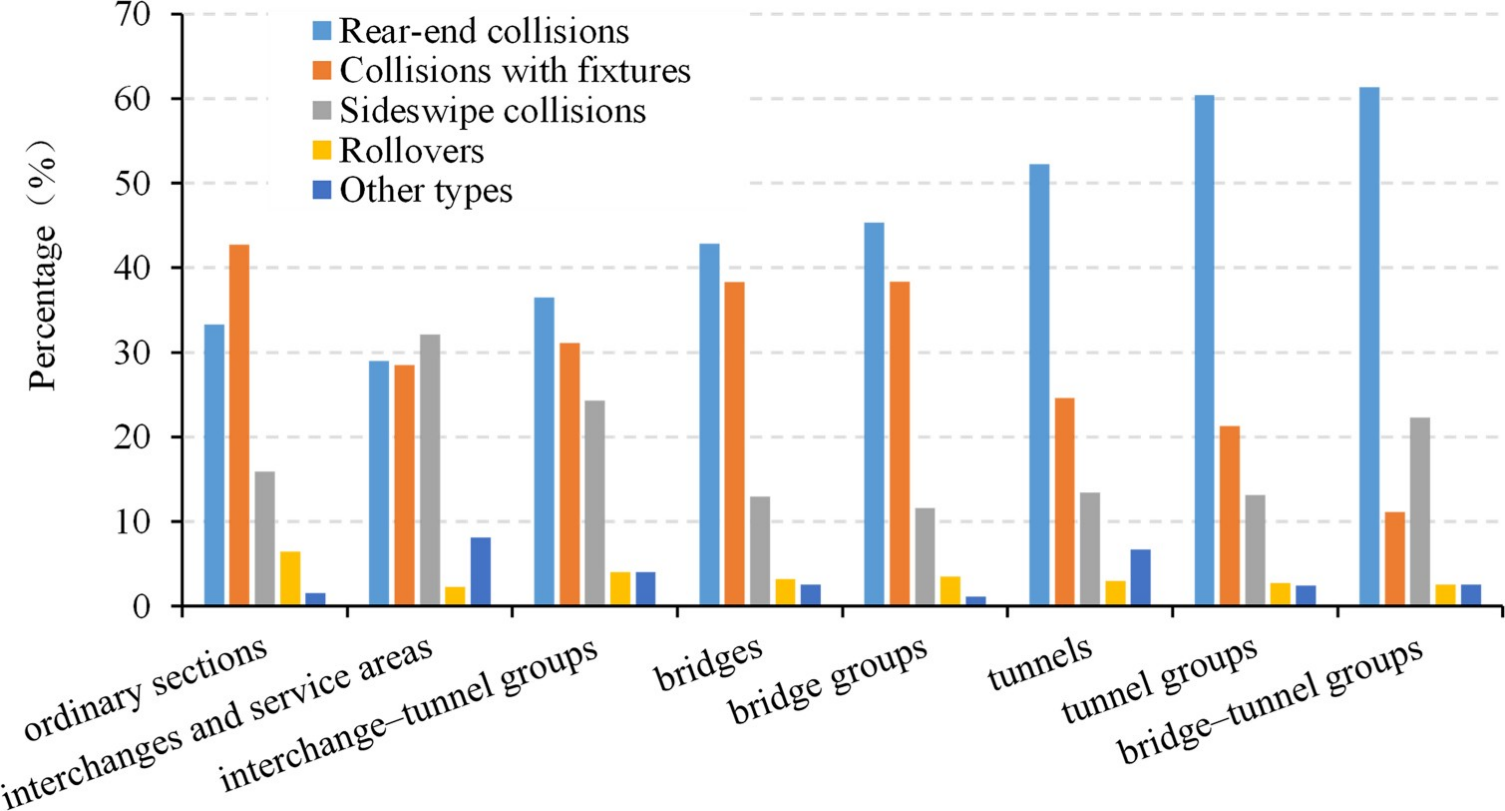
Distribution of crash types by road sections.

The distribution of crashes on each type of road section is shown in Fig 3. Rear-end collisions are the most frequent on most of the road sections, followed by collisions with fixtures and sideswipe collisions. However, on ordinary road sections, collisions with fixtures are more frequent than rear-end collisions owing to the higher speed of vehicles. Moreover, interchanges and freeway service areas have the highest rates of sideswipe collisions owing to the large number of lane-changing maneuvers. Accordingly, rollovers and other crashes occurred most frequently on ordinary roads as well as interchanges and service areas. The latter was also closely related to people–car conflicts, random parking, illegal parking, etc.

**Fig 4:**
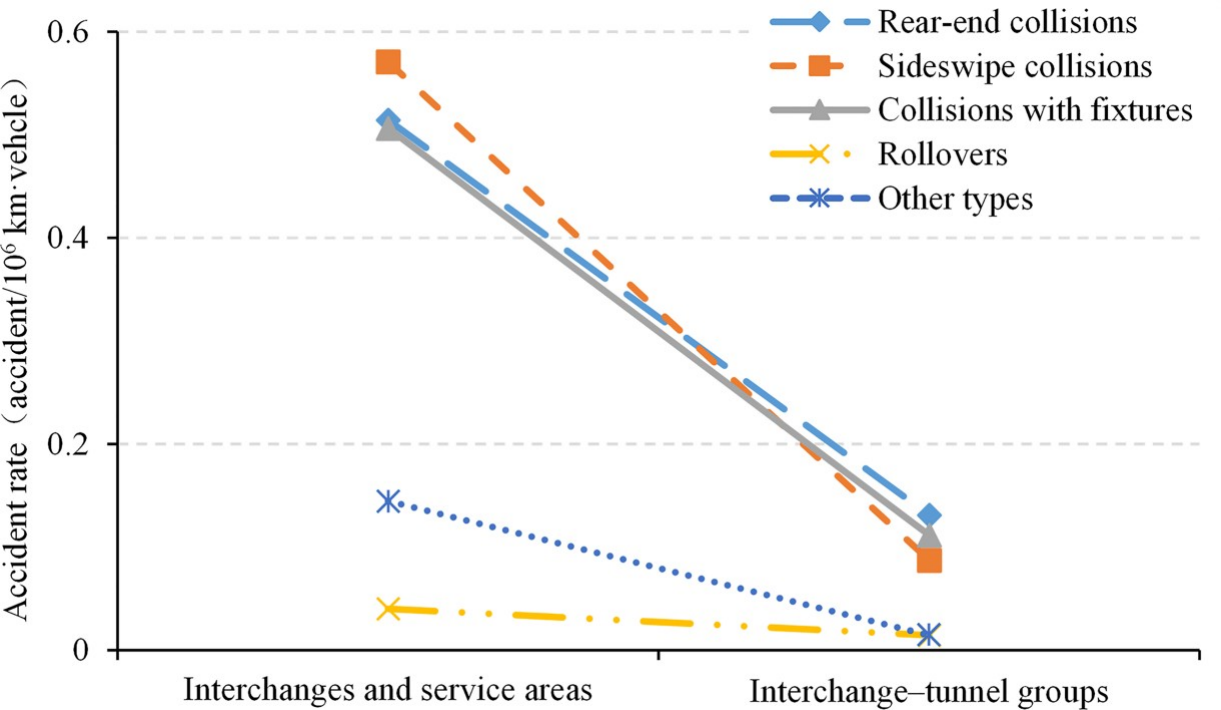
Distribution of crash rates on sections with special traffic organization.

**Fig 5:**
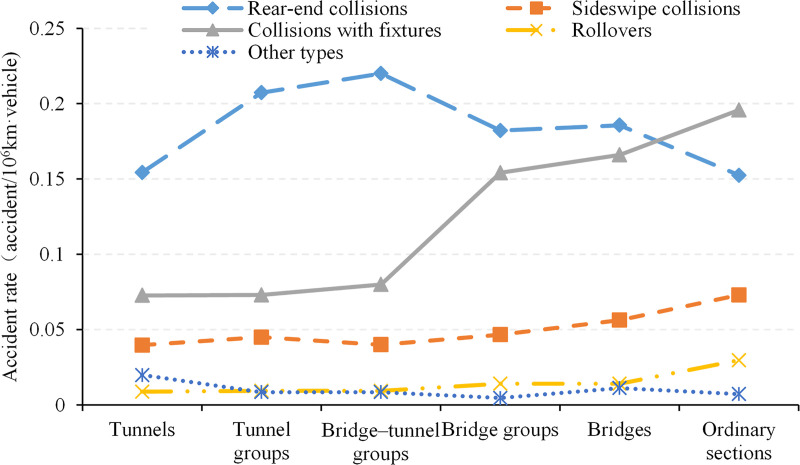
Distribution of crash rates on sections without special traffic organization.

To further explore the differences in crash rates on the different types of road sections, the crash rates were aggregated according to the collision type. Figs 4 and 5 show the breakdown of crashes into five crash types. Fig 4 shows that on road sections with unique traffic organization, the crash rates of sideswipe collisions are significantly higher than those on the other sections and are similar to the levels of rear-end collisions and collisions with fixtures. Most crashes were caused by these three types of collision. On interchanges and at freeway service areas, all the crash rates exceeded 0.5. Thus, the overall crash rates on interchanges and at service areas were significantly higher than that on other sections. In contrast, the rear-end crash rate of interchange–tunnel groups was similar to that of other sections, and sideswipe collisions and collisions with fixtures were the most frequent causes of crashes.

As shown in Fig 5, on road sections without unique traffic organization, the rates of sideswipe collisions, rollovers, and other crashes were rather low and stable across different road sections, and the former is higher than the latter two, whose averages were 0.05 and 0.01, respectively. Meanwhile, the rate of rear-end crashes remained high, especially on the unique road sections with composite structures. However, the overall difference was still relatively small (between 0.15 and 0.20), and the corresponding crash rate of collisions with fixtures sharply increased from 0.07 to 0.20. Thus, the risk of rear-end collision remained high on these sections; however, with decreasing scene action intensity and increasing vehicle speed, collisions with fixtures were the main reason for the difference in crash rates. In particular, safe driving declined most prominently on ordinary sections, bridges, and bridge groups.

### Crash severity

The severity of traffic crashes is an important aspect that needs to be considered. In this study, a total of 1739 crashes were analyzed, including 26 with deaths, 68 with injuries, and 1,645 with only property damages. These crashes resulted in 285 casualties. The severity of the crashes among the various road sections is detailed in Table 5. In the event of a crash, deaths occurred in 1.5% of the crashes on the road section, whereas the corresponding percentage on ordinary road sections was 1.23%. The probability of death in crashes was slightly higher in the former case than in the latter case. Furthermore, the fatality rates of injuries on the entire section and ordinary sections were 18.25% and 13.92%, respectively; the corresponding numbers of injuries per crash were 3.03 and 2.47, respectively. The two indexes of the entire section were significantly higher than those of ordinary sections. Thus, although the casualty rates between the two roads were generally similar (5.41% and 5.61%), the crash severity on the entire road was significantly higher than that on ordinary road sections owing to the unique road traffic scenes.

**Table 5:** Number of crashes by severity.

Crash severity	Fatalities		Injuries		Property Damage only	Total	
	Number of crashes	Number killed	Number of crashes	Number injured	Number of crashes	Number of crashes	No Killed or injured
Tunnels	2	4	7	14	125	134	18
Bridges	7	24	9	99	138	154	123
Interchanges and service areas	3	3	6	6	212	221	9
Tunnel groups	4	5	11	23	389	404	28
Bridge groups	3	5	4	15	79	86	20
Bridge-tunnel Groups	2	3	22	30	442	466	33
Interchange-tunnel groups	0	0	2	2	72	74	2
Ordinary sections	7 (1.23%)	11 (13.92%)	25 (4.38%)	68	539	571	79
Entire road section	26 (1.50%)	52 (18.25%)	68 (3.91%)	233	1645	1739	285

**Fig 6:**
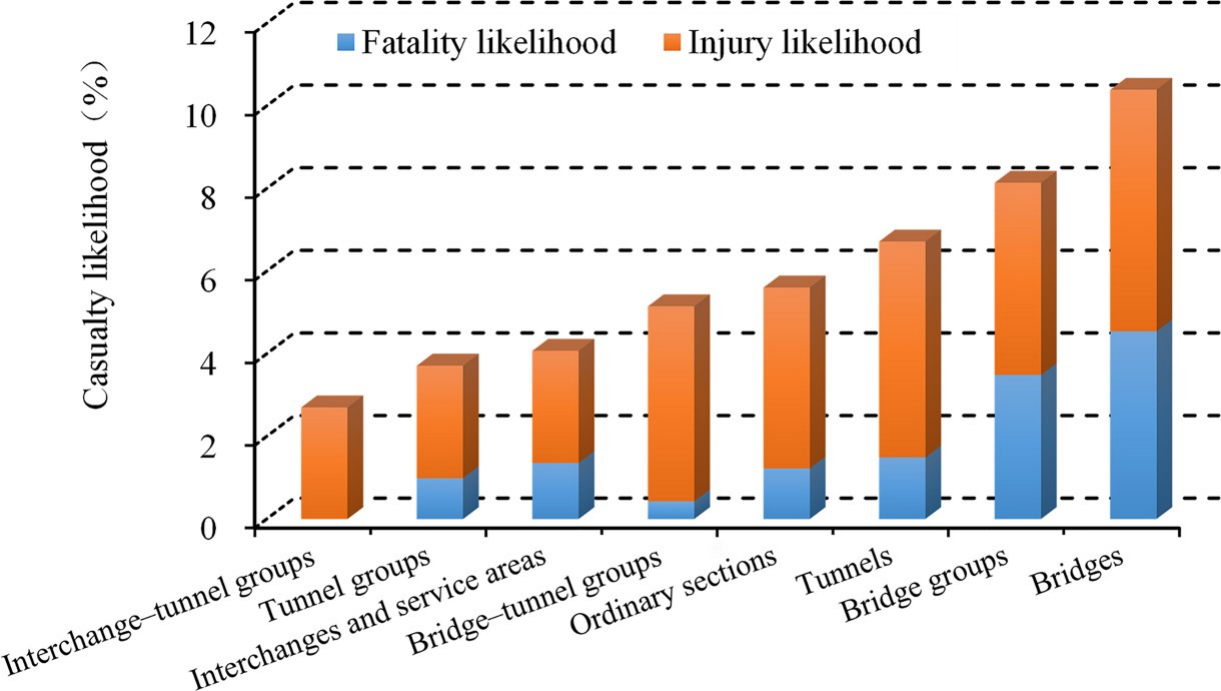
Casualty likelihoods on the various types of road sections.

Fig 6 indicates that the most severe injuries occurred on bridges, bridge groups, and tunnels, with casualty likelihoods of 10.39%, 8.14%, and 6.72%, respectively. Interchange–tunnel groups had the lowest proportion of injuries. In decreasing order, ordinary sections, bridge–tunnel groups, interchanges and freeway service areas, and tunnel groups had intermediate casualty likelihoods. Based upon crash seventy, the road sections can be ranked as follows: bridges > bridge groups > tunnels > ordinary sections > bridge–tunnel groups > interchanges and service areas > tunnel groups > bridge-interchange groups. In terms of the fatality rates alone, i.e., the likelihood that a traffic crash will involve deaths, similar patterns can be observed when compared with the likelihood of casualties. The only difference was that no deaths occurred in interchange–tunnel groups, and bridge–tunnel groups had the lowest number of deaths.

**Fig 7:**
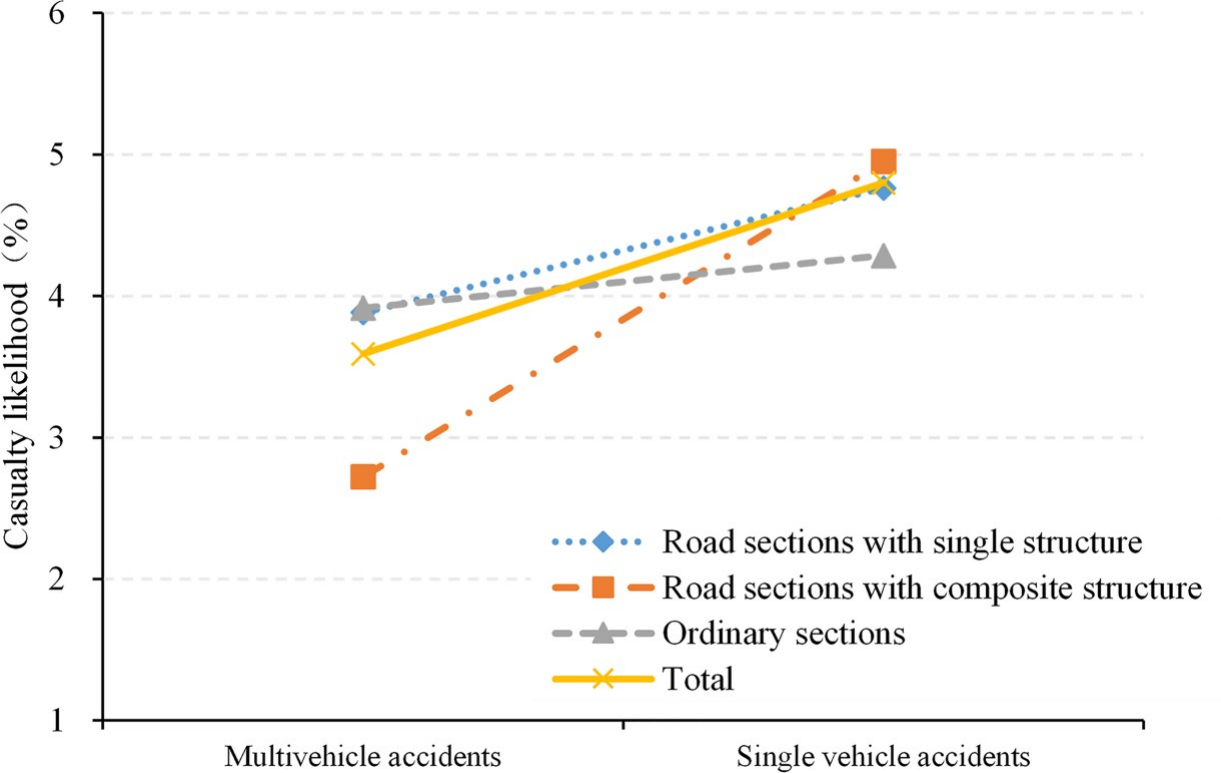
Casualty likelihoods of single-vehicle and multi-vehicle crashes.

Additionally, crash severities exhibit an inconsistent pattern with the crash rates. This difference can be explained by the proportion of single-vehicle crashes across the various road sections. As shown in Fig 7, the casualty rates of single- and multi-vehicle crashes were 4.8% and 3.59%, respectively. The former was approximately 1.4 times higher than the latter and thus had significantly more serious consequences. Therefore, when the proportion of single-vehicle crashes increases, the corresponding severity of road crashes increases.

### Crashes and AADT

**Fig 8:**
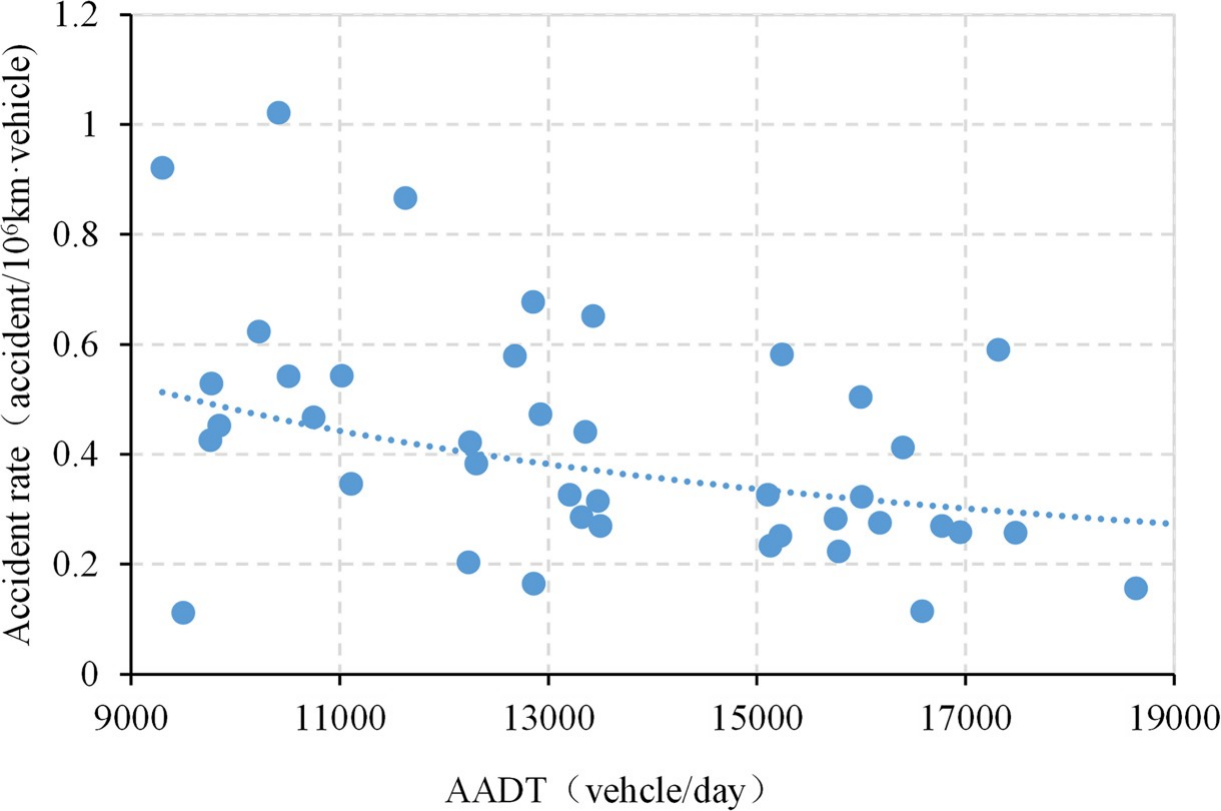
Correlation between AADT and crashes.

To investigate the relationship between traffic crashes and traffic volumes, AADT and the number of crashes per year between 2014 and 2016 are collected, and the corresponding crash rate calculation results are presented in Fig 8. As shown in the figure, crash rates decline slowly with increase in AADT. The result is consistent with other types of roads—the growth rate of crashes is lower than that of AADT. Hence, AADT is one of the important factors affecting crashes on mountainous freeways with high bridge and tunnel ratios.

### Crashes and driving adaptability

**Fig 9:**
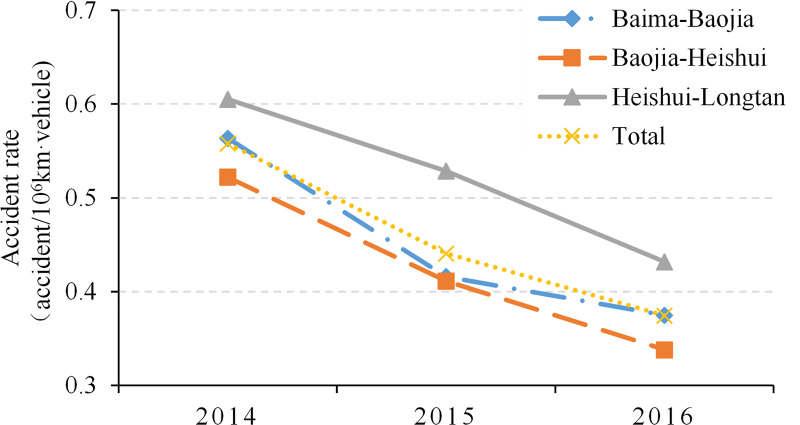
Correlation between time from roadway opening and traffic crashes.

The entire road section was divided into three typical sections: Baima-Baojia, Baojia-Heishui and Heishui-Longtan sections. The bridge and tunnel ratios of these three sections are close to 80%, 60%, and 50%, respectively, which are typical road sections with ultra-high, higher, and high bridge and tunnel ratios. It is worth noting that since all sections were opened to traffic by the end of 2010, the daily average traffic volume has been close to the design level of early 2014 after about three years of initial operation. In the following three years, no major safety improvement projects have been carried out. The corresponding crash rates are shown in Fig 9.

As shown in Fig 9, from 2014 to 2016, the crash rates of all three sections decreased significantly with increase in the length of time from the opening of the road. An increase in traffic volume during this time would likely have had an impact on the decline in crashes, but as the volume remained relatively constant, the decrease in crashes was likely attributable to the increasing adaptability of drivers to the road environment. In addition, it was found that the three sections show the same degree of decline in crash rates at different bridge and tunnel ratio levels during this period, all of which are approximately 0.18. This indicates that the driver’s adaptation process does not lead to any clear difference towards a reduction in crash rate for road sections with different bridge and tunnel ratios.

## Conclusions

As a road type that is attracting interest in China, mountainous freeways with high bridge and tunnel ratios contain a large number of structures, such as bridges, tunnels, and interchanges, which form many unique road sections. To investigate the characteristics of traffic crashes on these roads, a road discretization method based on structure scenes was established according to structure types. For various elementary structures, the traffic influence areas were defined by analyzing the safety characteristics of the entrances and exits. Using the proposed safety-critical distance index, we conducted quantitative determination and scene divisions of special sections for composite structures. Although this method is based on structures, it was not limited to the body of the structure but considered the scope of influence of one section on contiguous sections to improve the road discretization process and provide a good basis for research concerning crashes. This study investigated the crash data of a typical mountainous freeway. The results showed that this new type of roadway exhibits distinct crash characteristics and unique safety performance under the action of the special road traffic scene.

The road section types were ranked according to their crash rates as follows: interchanges and service areas > ordinary sections > bridges > bridge groups > bridge–tunnel groups = bridge-interchange groups > tunnel groups > tunnels. After disaggregation by crash types, the crash rates at interchanges and service areas were found to be remarkably higher than that in other road sections owing to the high crash rate of sideswipe collisions, rear-end collisions, and collisions with fixtures. In the other road sections, the rates of sideswipe collisions, rollovers, and other crashes were typically stable at a low level, whereas the rate of rear-end collisions was stable at a high level. Therefore, the differences in crash rates on different sections were mainly due to collisions with fixtures.

Multi-vehicle crashes accounted for a large proportion of crashes on the entire road section and for approximately 60% of all crashes in specific road sections. Moreover, rear-end collisions were the most frequent, which indicates that drivers encounter greater difficulty in dealing with interactions between vehicles. The crash severity was significantly lower in ordinary road sections than in the entire road section, especially in bridges, bridge groups, and tunnel groups. More specifically, a different pattern was found between crash rates and crash severity. This might be a result of the different proportions of single vehicle crashes, which were nearly 1.4 times more likely to lead to casualties than multi-vehicle crashes. The crash rate was found to decrease when AADT increased. Furthermore, the crash rate decreased with increase in the road opening time, which suggests that the drivers’ adaptability is enhanced in this process.

The results demonstrate the road design and traffic management implications. First, safety improvements should be implemented, particularly in interchanges and service areas because they have the highest crash rates. In addition, considering that it is difficult to select interchange and service area sites because of the complex terrain conditions in mountainous areas, they should be designed as far apart as possible or combined such that their numbers are reduced. For example, in the freeway section previously studied, the Wulong service area and interchange were designed as a whole for both areas to be combined at one location to reduce the number of complex driving situations in that area. Second, owing to the high crash rate, general open road sections (e.g., ordinary sections and bridge sections) should be more carefully designed, focusing on road alignment. Generally, because the complex alignment of mountainous freeways with high bridge and tunnel ratios cannot be realized in tunnels with high alignment requirements, most of the open sections inevitably become alignment adjustment sections or special sections such as sections with long and steep slopes and small radius curves. Therefore, the road alignment of these sections is complex, which results in frequent crashes. Third, on a whole, frequent multi-vehicle crashes indicate that driver assistance systems may be an effective solution to solve this problem. Meanwhile, for general open sections, the high number of single vehicle crashes shows that speed management systems such as variable speed systems need to be considered. These systems have delivered promising results in reducing crashes [24–26]. Finally, the overall safety of the road is closely related to the combination design of bridges and tunnels, and traffic management needs to be strengthened particularly in the early stage of road operation.

Several limitations should be noted for this study. Because of the limitation of crash data sources, this study only analyzes crashes that occurred between 2014 and 2016 on a mountainous freeway with a high bridge and tunnel ratio. Mountainous freeways with high bridge and tunnel ratios are unique roads with many road environments and complex geometrical road alignments, in which a crash is a result of the coupling effect of the two factors [17]. However, this study did not consider the effects of road alignment or external environmental factors such as weather, season, and time of day. It is also worth noting that the crash data were obtained from the police reports and may thus be subject to some degree of under-reporting. This fact is particularly true in China [27]. With the help of more data sources, future studies using statistical analytical methods should be conducted to analyze how these factors interact with each other in order to better reveal the casual mechanism underlying crashes on the road. Moreover, similar to the study conducted by Xu et al. [28], uncertainty analysis on indicators, such as crash rate and casualty rate, are needed to understand their statistical characteristics.
